# Diagnostic performance of CT lung severity score and quantitative chest CT for stratification of COVID-19 patients

**DOI:** 10.1007/s11547-022-01458-9

**Published:** 2022-02-14

**Authors:** Damiano Caruso, Marta Zerunian, Michela Polici, Francesco Pucciarelli, Gisella Guido, Tiziano Polidori, Carlotta Rucci, Benedetta Bracci, Giuseppe Tremamunno, Andrea Laghi

**Affiliations:** Department of Medical Surgical Sciences and Translational Medicine, Sapienza University of Rome, Sant’Andrea University Hospital, Via di Grottarossa, 1035-1039, 00189 Rome, Italy

**Keywords:** COVID-19, Lung quantification, Severity stratification, Chest CT, Lung severity score

## Abstract

**Purpose:**

Lung severity score (LSS) and quantitative chest CT (QCCT) analysis could have a relevant impact to stratify patients affected by COVID-19 pneumonia at the hospital admission. The study aims to assess LSS and QCCT performances in severity stratification of COVID-19 patients.

**Materials and methods:**

From April 19, 2020, until May 3, 2020, patients with chest CT suggestive for interstitial pneumonia and tested positive for COVID-19 were retrospectively enrolled and stratified for hospital admission as Group 1, 2 and 3 (home isolation, low intensive care and intensive care, respectively). For LSS, lungs were divided in 20 regions and visually assessed by two radiologists who scored for each region from non-lung involvement as 0, < 50% assigned as 1 and > 50% as 2. QCCT was performed with a dedicated software that extracts pulmonary involvement expressed in liters and percentage. LSS and QCCT were analyzed with ROC curve analysis to predict the performance of both methods. *P* values < 0.05 were considered statistically significant.

**Results:**

Final population enrolled included 136 patients (87 males, mean age 66 ± 16), 19 patients in Group 1, 86 in Group 2 and 31 in Group 3. Significant differences for LSS were observed in almost all comparisons, especially in Group 1 vs 3 (AUC 0.850, *P* < 0,0001) and Group 1 + 2 vs 3 (AUC 0.783, *P* < 0,0001). QCCT showed significant results in almost all comparisons, especially between Group 1 vs 3 (AUC 0.869, *P* < 0,0001). LSS and QCCT comparison between Group 1 and Group 2 did not show significant differences.

**Conclusions:**

LSS and QCCT could represent promising tools to stratify COVID-19 patient severity at the admission.

## Introduction

Since severe acute respiratory syndrome coronavirus 2 (SARS-CoV-2) has been reported as cause of a new viral pneumonia coronavirus disease 2019 (COVID-19) in Wuhan, Hubei, China, in December 2019, the next weeks spreading has led a pandemic diffusion of the virus all over the world, with over 58 million infected people, and the number is still increasing [1].

Awaiting specific treatments and vaccine effects, the best approach consists in early diagnosis, correct severity stratification and supportive therapy to allow a better prognosis in critical patients [2]. Some clinical and laboratory biomarkers are emerging as predicting tools to help physicians in the correct stratification of patients [3–5]. Among possible severity biomarker, chest computed tomography (CT) represents a valid noninvasive option [5–8]. Chest CT has shown high sensitivity (97%) despite low specificity (25–56%) for the diagnosis of COVID-19 [9, 10]. In addition, chest CT allows the assessment of COVID-19 lung impairment, mostly represented by multiple and peripheral ground-glass opacities (GGO) and possible associated consolidations [10, 11]. These alterations correlate with lung function in patients affected by acute respiratory disease syndrome (ARDS) [12], and COVID-19 could get worse until a severe lung injury with ARDS and need of intubation. Since now, timeliness in recognizing lung impairment is essential to change supportive therapy to have a better outcome [13].

Some authors proposed different lung severity scores [13–16] achieved by a visual assessment of the pulmonary impairment. Lung severity scores could have an impact in the clinical management to stratify patients and guide the clinical management at the admission and during follow-up [16, 17] despite intrinsic limitations of visual assessment such as poor reproducibility in clinical contest and lack of standardization of different proposed methods.

To overcome visual assessment limitations, new imaging tools are emerging; an example is represented by deep-learning pulmonary quantification proposed by Huang et al. [18] or CT-aided quantification software [19]. Since now, a few studies have correlated quantitative lung impairment with clinical assessment and follow-up, but more studies are needed to confirm the reliability of lung quantification in clinical set.

Thus, the aim of our study is to assess the performance of chest CT in severity stratification of COVID-19 patients at the hospital admission and to compare diagnostic performances of lung severity score (LSS) and quantitative chest CT (QCCT).

## Methods and materials

### Patient population and admission groups

This study was approved by our local institutional review board (IRB) and conducted in accordance with the Declaration of Helsinki. Informed consent was obtained from all patients, when patients were in a condition of inability their relatives or the admitting physicians provided it. Four-hundred-seven consecutive patients admitted at the Emergency Department of BLINDED with interstitial pneumonia from April 19, 2020, until May 3, 2020, were retrospectively included in the study.

Inclusion criteria were: (1) patients admitted to emergency department with suspicion of COVID-19, (2) patients with highly suspected chest CT for interstitial pneumonia, according to the main lung features linked to typical COVID-19 pneumonia [10, 11]. Exclusion criteria were: (1) patients tested with swabs for SARS-CoV-2 detection and resulted negative, (2) patients who underwent chest CT with contrast medium injection, (3) who refused chest CT, (4) patients with history of lung malignancy that required pulmonary resection and (5) chest CT with deteriorated images from motion artifact.

According to the hospital internal protocol, when suspected COVID-19 patients were admitted presenting moderate–severe clinical features and a high pretest probability of disease (fever defined as > 37.5 °C and respiratory symptoms or direct contact with a confirmed COVID-19 patient) underwent nasopharyngeal and oropharyngeal swabs for SARS-CoV-2, and chest CT to assess lung impairment, chest CT was performed at the entrance, to have a real-time evaluation of lung parenchyma at baseline.

Every patient was tested with two nasopharyngeal and oropharyngeal swabs, the first swab at the entrance and the second after 24 h. The positivity to SARS-CoV-2 was obtained with reverse transcriptase-polymerase chain reaction (RT-PCR) (Charitè, Berlin, Germany) [20], while patients were considered SARS-CoV-2 negative after two consecutive negative RT-PCR results. For all included patients, demographic data and laboratory results were collected (**Table ****1**).

**Table 1:** Patients demographics, clinical and laboratory tests characteristics

Mean age (range)	Group 1		Group 2		Group 3		Group 1 + 2		Group 2 + 3	
	65 ± 15 (36–90)		61 ± 14 (33–92)		71 ± 14 (28–97)		64 ± 15 (33–92)		62 ± 14 (28–97)	
Patients demographics	No. of patients	%	No. of patients	%	No. of patients	%	No. of patients	%	No. of patients	%
Number of patients	19	100	86	100	31	100	105	100	117	100
Male	15	79	54	63	20	65	69	66	74	63
Female	4	21	32	37	11	35	36	34	43	37
*Blood test*										
C-reactive protein (mg/L; normal range 0.0–5.0)										
Increased	16	84	76	88	31	100	92	88	107	92
Normal	3	16	10	12	0	0	13	12	10	8
Lactic acid dehydrogenase (U/L; range 125–220)										
Increased	11	58	64	74	28	90	75	71	92	79
Normal	8	42	22	26	3	10	30	29	25	21
Lymphocytes (× 10^3^/mm^3^, normal range 1.5–3.0)										
Increased	0	0	2	2	1	3	2	2	3	3
Decreased	14	74	65	76	25	81	79	75	90	77
Normal	5	26	19	22	5	16	24	23	24	20
D-dimer (ng/ml, normal < 243)										
Increased	8	42	46	53	27	87	54	51	73	62
Normal	11	58	40	47	4	13	51	49	44	38
*Symptoms*										
Fever (> 37.5 °C)	2	11	30	35	16	52	32	30	46	39
Cough	7	37	39	45	16	52	46	44	55	47
Dyspnea	5	26	20	23	17	54	25	24	37	32
Positive link	4	21	27	32	9	29	31	30	36	31

In addition, at the hospital admission, patients were clinically stratified for severity of symptoms and care necessity in home isolation or hospitalization (low intensive or intensive care) according to the guidelines of our hospital [21], then the population was divided in 3 Groups: Group 1 home isolation, Group 2 low intensive care and Group 3 intensive care.

### CT acquisition technique

All suspected COVID-19 patients underwent chest CT to evaluate the presence of interstitial pneumonia. Chest CT was acquired without contrast medium and in supine position during end-inspiration. Each patient was studied using a COVID-19 dedicated 128-slice CT (GE Revolution EVO 64 Slice CT Scanner, GE Medical Systems, Milwaukee, WI, USA). CT scan technical parameters were as follows: tube voltage: 100 kV; tube current modulation 100–250 mAs; Asir-V 50%, spiral pitch factor: 0.98; collimation width: 0.625. Reconstruction images were performed with convolution kernel BONEPLUS at a slice thickness of 1.25 mm.

### Lung severity score

A visual assessment of lung COVID-19 impairment was performed by two radiologists in consensus (GG and DC with 6 and 8 years of experience). According to lung severity score (LSS) already proposed in the literature [9], lungs were divided in 20 regions; the number of 20 was reached starting from the anatomical division in 18 lung segments, two of which were further divided in two regions: the anterior medial basal segment of the inferior left lobe was split in anterior basal and medial basal while the posterior apical segment of the superior left lobe was divided into apical and posterior regions.

To each segment, readers were assigned a visual percentage of parenchymal involvement (including GGO, consolidation and pleural effusion) scoring from a non-involvement expressed with 0, less than 50% of involvement assigned as 1 and a score of 2 was given for more than 50% of lung involvement. Thus, with the maximum score reachable of 40, readers made a visual assessment of all selected patients, on the reconstructed images, with possibility of multi-planar reconstruction and a fixed window level set for lung (WW/LL:1600/-600 HU).

### Quantitative chest CT

Two radiologists in consensus (FP and MP with 5 and 4 years of experience), blinded to clinical patients’ stratification, performed QCCT analysis by using a dedicated software (Thoracic VCAR v13.1, GE). Before segmentation, attenuation value < − 1000 HU was used to exclude trachea air from the analysis. Quantitative analysis was performed on naive acquisition using a lung window with a width of 1500 HU and a level of − 600 HU, in particular the selection of well-aerated lung was performed by using a range between − 950 and − 700 HU density [22–24]. The software automatically calculated the following features: GGO, consolidation, fibrotic-like alterations (including fibrotic-like streaks and subpleural lines), total lung impairment and healthy lung, using an adaptive mean based on gray scale, expressed in percentages. Vessel was automatically selected and delated. In case of non-adequate automatic segmentation, readers were free to adjust the area of lung impairment segmented by the software [25].

### Statistical analysis

Statistical analysis was performed using MedCalc Statistical Software version 17.9.7 (MedCalc Software bvba, Ostend, Belgium), and *P* values < 0.05 were considered statistically significant. All data are expressed as mean ± standard deviation (SD). Kolmogorov–Smirnov test was used to assess data distribution. In case of Gaussian distribution, data were tested with Student’s *t* test, while Wilcoxon test was applied for non-Gaussian distributed data.

LSS and QCCT were analyzed with receiver operating characteristic (ROC) curves and the area under the curve (AUC) was calculated for predicting the performance of both methods for distinguishing clinical stratified patients at the hospital admission Group 1, 2 and 3. Further sub-analysis was performed comparing performance of LSS and QCCT for Group 1 and together Group 2 + 3 and between Group 1 + 2 against Group 3. For LSS, the interobserver agreement was also evaluated.

## Results

### Patient population

**Fig. 1:**
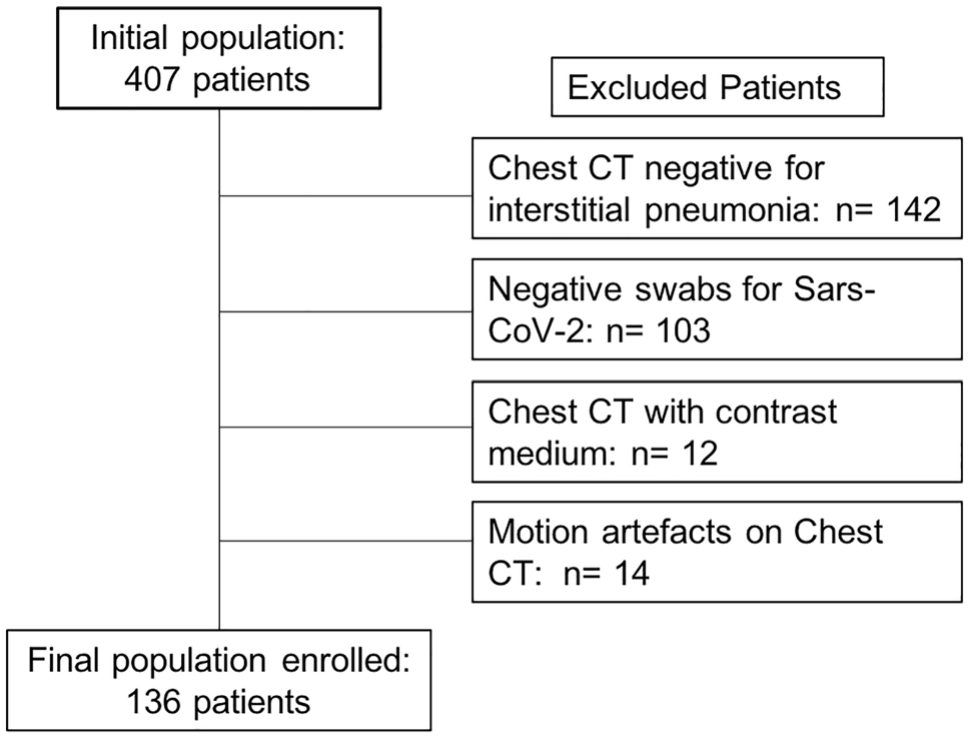
Patients’ enrollment flowchart

According to exclusion criteria, from the initial population of 407 patients, were excluded: 142 patients due to negative chest CT for interstitial pneumonia, 103 patients negative for SARS-CoV-2 swabs, 12 patients who underwent chest CT with contrast medium injection and 14 patients for motion artifacts on chest CT (Fig. 1). Final population enrolled included 136 patients, 87 male 49 female (mean age 66 ± 16, range 28–97); in particular, 19 patients belonged to admission Group 1, 86 were in Group 2, while 31 were admitted in Group 3. Full clinical data divided per admission groups are displayed in Table 1.

Significant differences were observed in C-reactive protein and D-dimer between Group 1 and 3 with *P* = 0.0058 and *P* = 0.0005; lactic acid dehydrogenase and D-dimer had significant differences between Group 2 and Group 3 with *P* = 0.0006 and *P* = 0.0007, respectively. Group 1 + 2 compared to Group 3 showed significant differences for C-reactive protein, lactic acid dehydrogenase and D-dimer with *P* = 0.0006, *P* = 0.0005 and *P* = 0.0001, respectively. No significant differences among other clinical parameters were observed.

### Lung severity score

Chest CT LSS showed significant differences for Group 1 compared to Group 3 (AUC 0.850, sensitivity 51.61%, specificity 100%, cutoff > 22, *P* < 0.0001), Group 2 vs Group 3 (AUC 0.768, sensitivity 74.19%, specificity 66.28%, cutoff > 17, *P* < 0.0001), Group 1 + 2 vs Group 3 (AUC 0.783, sensitivity 74.19%, specificity 67.62%, cutoff > 17, *P* < 0.0001) and Group 1 vs Group 2 + 3 (AUC 0.668, sensitivity 80.34%, specificity 52.63%, cutoff > 10, *P* = 0.0102). No significant differences were observed between Group 1 and 2 (*P* = 0.67). LSS interobserver agreement revealed an excellent value of 0.91.

### Quantitative chest CT

**Fig. 2:**
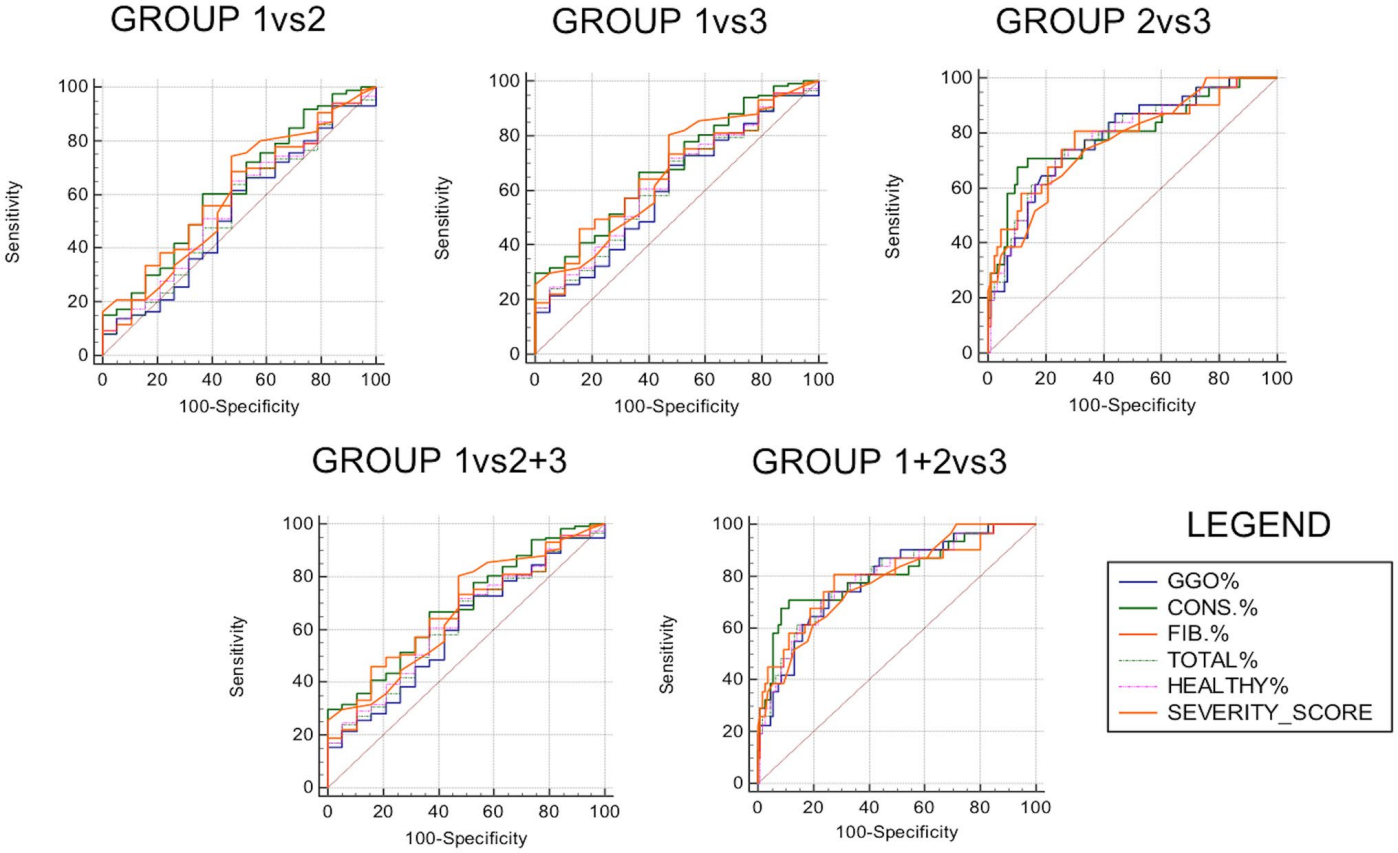
ROC curve of all features divided for all admission groups. Legend abbreviations: ground-glass opacities (GGO), consolidations (CONS.), fibrotic-like alterations (FIB.), total lung impairment (TOTAL), healthy parenchyma (HEALTHY) and lung severity score (SEVERITY_SCORE)

**Fig. 3:**
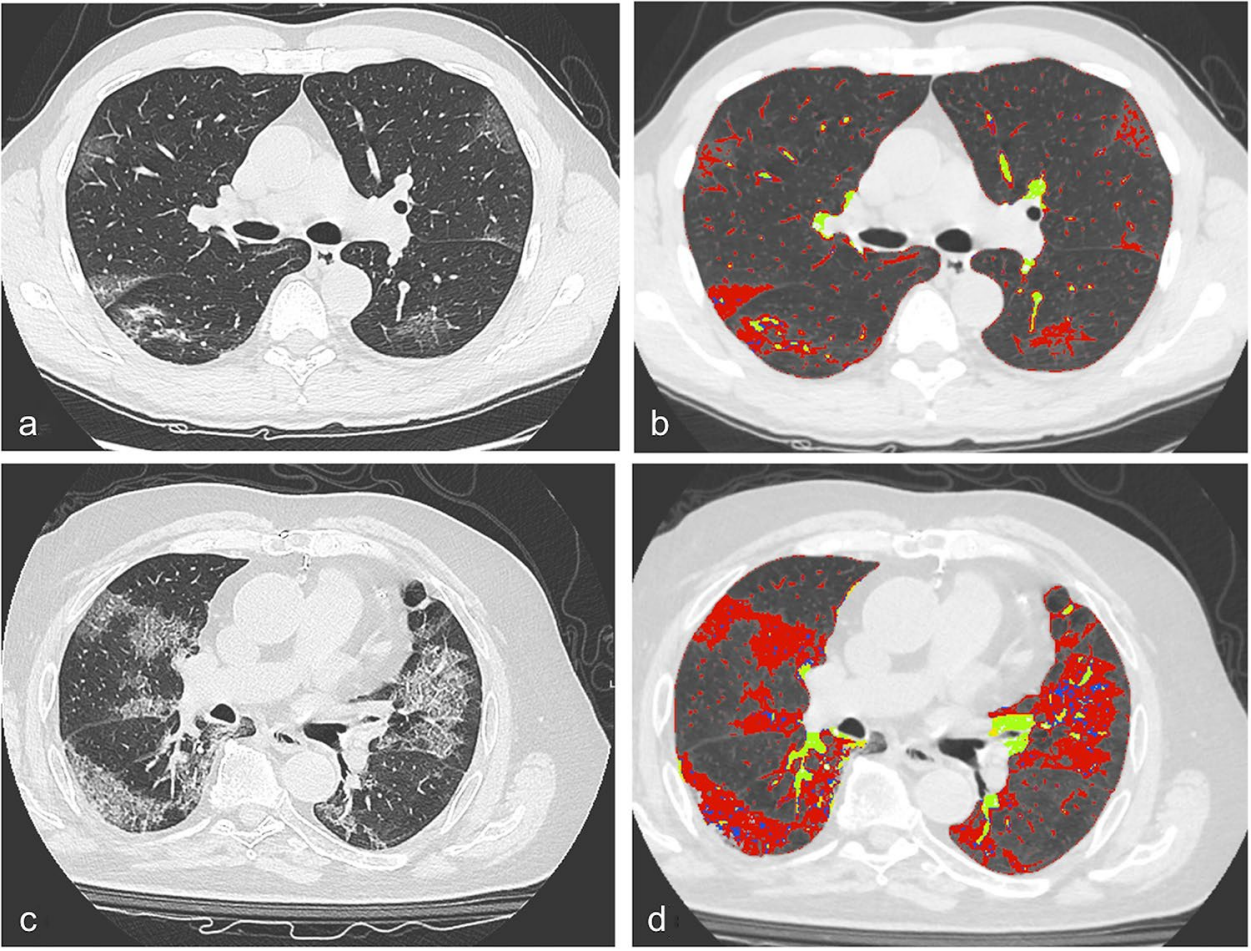
**a** Chest CT scan of a 69-year-old male patient followed at home isolation (Group 1) and **b** segmented in red corresponding quantitative chest CT reporting percentage of ground-glass opacities (9%), consolidations (1,19%), fibrotic-like alterations (0,85%), total lung impairment (11,06%) and healthy parenchyma (88,11%), while vessels in yellow were excluded from the semiautomatic analysis; patient’s lung severity score was evaluated with a score of 16. **c** A chest CT scan of an 83-year-old male patient in intensive care (Group 3) with the following quantitative chest CT showed in **d**: ground-glass opacities (30,42%), consolidations (2,07%), fibrotic-like alterations (3,19%), total lung impairment (35,69%) and healthy parenchyma (62,18%); patient’s lung severity score was 28

Performance of QCCT showed significant results for Group 1 vs Group 3 where the best two were consolidation (AUC 0.869, sensitivity 70.97%, specificity 100%, *P* < 0.0001) and fibrotic-like alteration (AUC 0.842, sensitivity 80.65%, specificity 84.21%, *P* < 0.0001). Similar significant results were observed for Group 2 vs Group 3, the best two features were represented by consolidation (AUC 0.794, sensitivity 67.74%, specificity 89.53%, *P* < 0.0001) and total lung impairment (AUC 0.790, sensitivity 70.97%, specificity 76.74%, *P* < 0,0001). Consolidation and total lung impairment were the best features for the sub-analysis of Group 1 + 2 vs Group 3 (AUC 0.808, sensitivity 70.97%, specificity 88.57%, *P* < 0.0001 and AUC 0.798, sensitivity 70.97%, specificity 77.14%, *P* < 0.0001, respectively), while consolidation and fibrotic-like alteration were the best for Group 1 vs Group 2 + 3 (AUC 0.688, sensitivity 29.91%, specificity 100.00%, *P* = 0.0023) and fibrotic-like alteration (AUC 0.662, sensitivity 46.15%, specificity 84.21%, *P* = 0.0095). No significant differences were observed between Group 1 vs Group 2. Detailed results are reported in Table 2 and ROC curves are displayed in Fig. 2. An explicatory example of QCCT analysis is provided in Fig. 3.

**Table 2:** ROC curve analysis of both chest CT lung severity score (LSS) and quantitative chest CT (QCCT)

Groups	Variables	AUC	Sensitivity (%)	Specificity (%)	Cutoff*	*P* value
1 vs 2	LSS	0,532	61,63	52,63	> 9,00	0,67
	QCCT					
	GGO	0,532	61,63	52,63	> 9,00	0,67
	Consolidation	0,623	60,47	63,16	> 1,19	0,09
	Fibrotic-like alterations	0,598	68,60	52,63	> 0,72	0,17
	Total lung impairment	0,545	64,00	52,60	> 11,05	0,55
	Healthy lung	0,562	65,10	52,06	≤ 88,03	0,40
2 vs 3	LSS	0,768	74,19	66,28	> 17,00	**< 0,0001**
	QCCT					
	GGO	0,786	74,19	74,42	> 16,57	**< 0,0001**
	Consolidation	0,794	67,74	89,53	> 1,91	**< 0,0001**
	Fibrotic-like alterations	0,787	80,65	69,77	> 1,33	**< 0,0001**
	Total lung impairment	0,790	70,97	76,74	> 20,79	**< 0,0001**
	Healthy lung	0,782	70,97	76,74	≤ 77,65	**< 0,0001**
1 vs 3	LSS	0,850	51,61	100,00	> 22	**< 0,0001**
	QCCT					
	GGO	0,806	74,19	73,68	> 16,61	**< 0,0001**
	Consolidation	0,869	70,97	100,00	> 1,86	**< 0,0001**
	Fibrotic-like alterations	0,842	80,65	84,21	> 1,27	**< 0,0001**
	Total lung impairment	0,830	61,29	89,47	> 24,30	**< 0,0001**
	Healthy lung	0,830	61,29	89,47	≤ 72,43	**< 0,0001**
1 + 2 vs 3	LSS	0,783	74,19	67,62	> 17	**< 0,0001**
	QCCT					
	GGO	0,789	74,19	74,29	> 16,61	**< 0,0001**
	Consolidation	0,808	70,97	88,57	> 1,87	**< 0,0001**
	Fibrotic-like alterations	0,797	80,65	72,38	> 1,33	**< 0,0001**
	Total lung impairment	0,798	70,97	77,14	> 20,83	**< 0,0001**
	Healthy lung	0,790	70,97	77,14	≤ 77,65	**< 0,0001**
1 vs 2 + 3	LSS	0,668	80,34	52,63	> 10	**0,0102**
	QCCT					
	GGO	0,605	69,23	52,63	> 9,00	0,127
	Consolidation	0,688	29,91	100,00	> 1,86	**0,0023**
	Fibrotic-like alterations	0,662	46,15	84,21	> 1,27	**0,0095**
	Total lung impairment	0,620	70,94	52,63	> 11,05	0,070
	Healthy lung	0,633	71,79	52,63	≤ 88,03	**0,0432**

^*^ Cutoff values range from 0 to 40 for LSS and are expressed in percentage for QCCT

*LSS* Lung severity score, *QCCT* Quantitative chest CT, *GGO* Ground-glass opacity

significant *P* values were reported in bold font

## Discussion

Our study tested the performance of chest CT lung severity score, based on the evaluation of the lung parenchyma involvement expressed in terms of percentage without a specific analysis concerning the type of alterations (i.e., consolidation, pleural effusion and GGO) and quantitative chest CT, performed by using a dedicated software which semiautomatically quantified each parenchymal changes, in differentiating COVID-19 patients at the admission into three different groups: home isolation (Group 1), low intensive care (Group 2) and intensive care (Group 3). Both LSS and QCCT showed significant and good performance in stratifying the severity of COVID-19 patients at the admission at the Emergency Department, in particular these helped in the identification of Group 3, the intensive care patients. The best diagnostic performance, in terms of AUC obtained, for discriminating COVID-19 patients in home isolation (Group 1) vs COVID-19 patients in intensive care (Group 3) was reached by lung severity score with an AUC of 0.850 (*P* < 0.0001) and by quantitative chest CT for consolidations and fibrotic-like alterations with AUC of 0.869 and 0.842 (all *P* < 0.0001), respectively. LSS results were also supported by the evaluation of interobserver agreement, that was excellent. We did not perform a qualitative score for each type of alterations to reduce the bias, which it is usually correlated to visual assessment. Furthermore, in the analysis of clinical laboratory data, we obtained some consistent differences into several comparisons made, showing the higher value of C-reactive protein, D-dimer, lactic acid dehydrogenase in the patients with high-risk diseases, needed low and intensive care.

Similar results were obtained for discriminating COVID-19 patients in low intensive care (Group 2) vs COVID-19 patients in intensive care (Group 3) for both LSS (AUC 0.768, *P* < 0.0001) and QCCT in terms of consolidation (AUC 0.794, *P* < 0.0001) and total lung impairment (AUC 0.790, *P* < 0.0001). Grouping both COVID-19 patients in home isolation and low intensive care (Group 1 + 2) vs Group 3, LSS returned with AUC of 0.783 (*P* < 0.0001) while QCCT for consolidation and total lung impairment showed an AUC of 0.808 and 0.798, respectively (all *P* < 0.0001). Interestingly, no differences were obtained between Group 1 and 2 for both LSS and QCCT.

The first general consideration regards the similar performance of both methods to identify COVID-19 patients in intensive care against Group 1 and 2, and Group 1 + 2, despite theoretically a semi-automated quantification seems more accurate than a visual one; similar results were obtained by Cong S. and colleagues [19] in terms of correlation between lesion percentage scored by radiologists and the computer software.

Despite differences among a wide diversity of lung severity scores [14, 16, 19, 26], our lung severity score results are in line with the others present in the literature, expressing a similar trend in terms of higher lung impairment observed with worsening of COVID-19 clinical conditions. More comparable results can be made with Yang et al. [16] due to the similarity of score adopted: their score achieved an AUC of 0.892 with a sensitivity and a specificity of 83% and 94%, respectively, and a cutoff > 19.5 to differentiate mild cases from severe cases. Our sub-analysis of Group 1 compared with Group 2 + 3 showed a cutoff > 10 for the LSS with an AUC of 0.668, a sensitivity of 80.34% and a specificity of 52.63%; on the contrary similar cutoff was observed for the Group 2 vs Group 3 and for the sub-analysis Group 1 + 2 compared with Group 3. Some discrepancies about cutoff values can be explained with different clinical stratification guidelines at the Emergency Department. However, the intrinsic limitation of visual assessment and consequent difficulties in the comparison among lung severity scores cannot be disregarded.

On the other hand, QCCT with percentage quantification extracted through the aided semi-automated method, let us make some interesting considerations. Consolidations, fibrotic-like alterations and total lung impairment resulted in the most significant parameters in terms of performance achieved with ROC curves among the different groups comparison.

Our results showed higher prevalence of consolidations impairment in more severe patients; this aspect can be explained with the progression of lung injuries due to increasing infiltration of both pulmonary parenchyma and interstitial spaces, caused by alveolar inflammatory exudation, diffuse alveolar damage and necrotizing bronchitis due to viral invasion and inflammatory system reaction [27, 28]. Likewise, higher percentage of Total Lung Impairment can be explained with the concomitant different lung injuries in severe patients such as GGO, crazy paving, consolidations and fibrotic-like alterations that concur to increase the total amount of lung injuries [19, 27, 29, 30]. Conversely, data regarding fibrotic-like alterations are less in accordance with other studies [26, 27]; in fact, Ding and colleagues [26] observed on a cohort of 112 patients, that linear opacities were more frequent in patients during stage 4 and 5 corresponding to 15–28 days after the beginning of symptoms. Also Lyu et al. define pulmonary fibrosis as uncommon CT findings at the baseline chest CT for all groups analyzed divided for disease severity [27]. A possible explanation is that patients admitted at the hospital had pneumonia symptoms some days before they arrived at the Emergency Department and when they underwent chest CT, some of the alterations, such as secondary organizing pneumonia, might be compatible with a medium or late stage of pneumonia [31]. Interestingly, GGO percentage does not have a great performance to stratify patients, in accordance with Lyu P. and colleagues findings [27]. Finally, percentage healthy lung parenchyma cutoff between Group 1 in comparison with Group 3 (cutoff < 72%, AUC 0.83) is in accordance with the cutoff showed by Colombi D. and colleagues performed on 236 patients (cutoff < 71%, AUC 0.86) [23].

Moreover, our consistent differences in laboratory data between Group 1 and Groups 2–3 are in consensus with the previous study of Watanabe et al. [5], in which were demonstrated higher values of inflammation makers in patients needed intensive care. Then, inflammation markers could be associated with consistent parenchymal impairment, in a setting of cytokines storm.

Limitations of our study include the retrospective nature of it, the lack of clinical and radiological follow-up, absence of a combined clinical and radiological model for the patient’s stratification and the choice to use a CT-aided program for lung quantification instead of deep-learning model, lack of comparison between visual and quantitative score, patients enrolled in an early pandemic scenario in which the severity was extraordinarily high and it could represent a patient selection bias, lack of LSS interobserver agreement. In the future, we want to overcome these drawbacks with the aim to perform an analysis on a more heterogeneous population, by using a more consistent quantification software, and with some data of follow-up.

In conclusion, our study demonstrates the feasibility of both chest CT lung severity score and quantitative chest CT as tools to stratify COVID-19 patients severity at the Emergency Department admission; quantitative chest CT might be integrated with clinical parameters to help accurate triaging of COVID-19 patients.

## Data Availability

The datasets generated and/or analyzed during the current study are available from the corresponding author on reasonable request.

## Abbreviations

SARS-CoV-2: Acute respiratory syndrome coronavirus 2
COVID-19: Coronavirus disease 2019
CT: Computed tomography
GGO: Ground-glass opacities
ARDS: Acute respiratory disease syndrome
LSS: Lung severity score
QCCT: Quantitative chest CT
IRB: Local institutional review board
RT-PCR: Reverse transcriptase-polymerase chain reaction
ROC: Receiver operating characteristic
AUC: Area under the curve

## Code availability

Texture analysis was performed by using a dedicated software (TexRAD, Feedback Medical Ltd., Cambridge, UK). Statistical analysis was performed using MedCalc Statistical Software version 17.9.7 (MedCalc Software bvba, Ostend, Belgium).
